# Supplementary material for: Injectable Nanoparticle‐Based Hydrogels Enable the Safe and Effective Deployment of Immunostimulatory CD40 Agonist Antibodies
Source: Adv Sci (Weinh). 2022 Aug 17;9(28):2103677. doi: 10.1002/advs.202103677 (PMC9534946; doi:10.1002/advs.202103677)
Supplement: Supplementary file 1 — Supporting Information [file ADVS-9-2103677-s001.pdf]

Supplementary Materials

Injectable Nanoparticle-Based Hydrogels Enable the Safe and Effective Deployment of Immunostimulatory CD40 Agonist Antibodies

Santiago Correa[1], Emily L. Meany[2], Emily C. Gale[3], John H. Klich[2], Olivia M. Saouaf[1], Aaron T. Mayer[2], Zunyu Xiao[4], Celine Liong[2], Ryanne A. Brown[5], Caitlin L. Maikawa[2], Abigail K. Grosskopf[6], Joseph L. Mann[1], Juliana Idoyaga[7], Eric A. Appel[1, 2, 8, *]

1- Department of Materials Science & Engineering, Stanford University, Stanford, CA 94305, USA
2- Department of Bioengineering, Stanford University School of Medicine, Stanford, CA 94305, USA
3- Department of Biochemistry, Stanford University, Stanford, CA 94305, USA
4- Department of Radiology, Stanford University School of Medicine, Stanford, CA 94305, USA
5- Department of Pathology, Stanford University School of Medicine, Stanford, CA 94305, USA
6- Department of Chemical Engineering, Stanford University, Stanford, CA 94305, USA
7- Microbiology and Immunology
8- ChEM-H Institute, Stanford University, Stanford, CA 94305, USA
* To whom correspondence should be addressed; E-mail: eappel@stanford.edu

Table of Contents

Supplemental Figure 1. Batch-to-batch oscillatory and flow rheology…………………………...….p3
Supplemental Figure 2. Batch-to-batch summary rheology properties & self-healing……….......p4
Supplemental Figure 3. *In vitro* release behavior of CD40a from hydrogels……………………..p5
Supplemental Figure 4. Abscopal effects of locoregional CD40a delivery………………………..p5
Supplemental Figure 5. Representative PET images of CD40a biodistribution…………..…....p6
Supplemental Figure 6. CD40a release kinetics from injection site………………………..…...…p7
Supplemental Figure 7. PET values versus explanted gamma counter measurements….…….p7
Supplemental Figure 8. C_{max} and time-to-C_{max} values……………………..……………………..……p8
Supplemental Figure 9. Weight loss due to CD40a treatment……………………..………………p9
Supplemental Figure 10. Additional breakdown of CD40a-induced hepatotoxicity…………......p10
Supplemental Figure 11. Cleaved caspase-3 assay of liver tissue….……………………….....…p11
Supplemental Figure 12. CD40a induced splenomegaly………………..…………………….......p12
Supplemental Figure 13. CD40a induced lymph node expansion….………………………....…p13
Supplemental Figure 14. Blood chemistry following CD40a treatment.…………………………p14
Supplemental Figure 15. CD40a dose-sparing efficacy with hydrogel delivery……………...…p14
Supplemental Figure 16. Serum cytokine data shown in pg/mL…………………..…………...…p15
Supplemental Figure 17. Full artifact-corrected serum cytokine Luminex results…….……….p16
Supplemental Figure 18. Highlighted artifact-corrected serum cytokine data……….…..….….p17
Supplemental Figure 19. Linear regression of dose-serum cytokine response……………...…p18
Supplemental Figure 20. Serum cytokine hierarchical clustering…………………….…….…..…p19
Supplemental Figure 21. TdLN cytokine data shown in pg/mg total protein……….…..….....….p20
Supplemental Figure 22. Full artifact-corrected TdLN cytokine Luminex results …….………….p21
Supplemental Figure 23. Highlighted artifact-corrected TdLN cytokine data……….…..….…..…p22
Supplemental Figure 24. Linear regression of dose-TdLN cytokine response………………....p24
Supplemental Figure 25. TdLN cytokine hierarchical clustering……………………..…..…..….p25
Supplemental Figure 26. Transformation of tumor immune microenvironment………………...p26

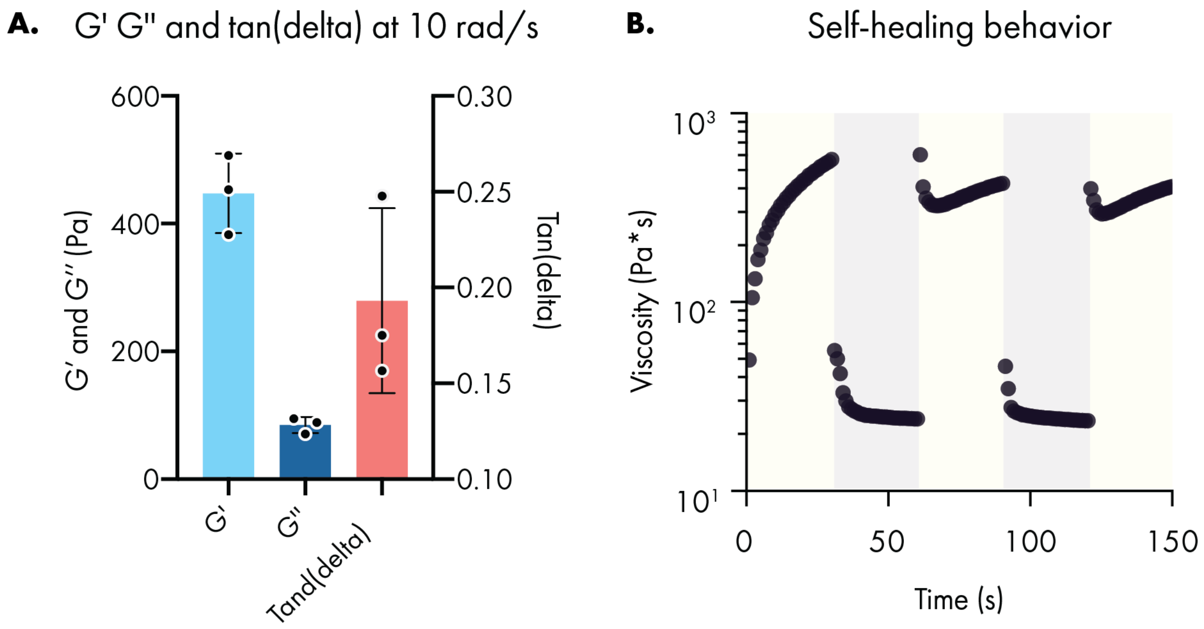

Supplemental Figure 1. Inter-batch variability of the rheological properties for PNP hydrogels and self-healing behaviors. (A) Independent batches of PNP hydrogel exhibit consistent moduli and solid-like properties. (B) Step-shear rheological behavior after two cycles of high shear (10 s-1) with intermediate low shear (0.1 s-1) cycles.

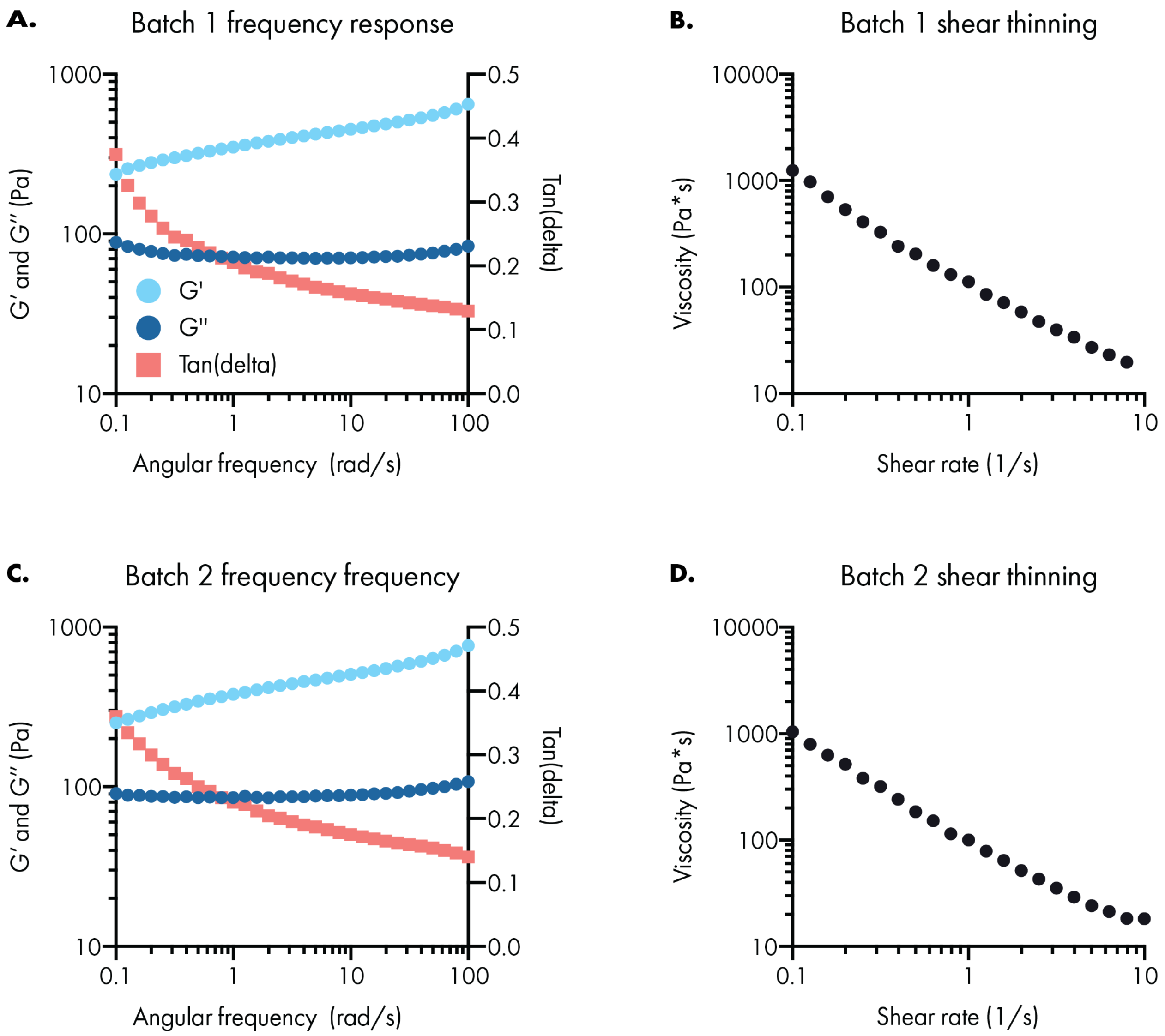

Supplemental Figure 2. Additional rheology data for replicate PNP hydrogels.

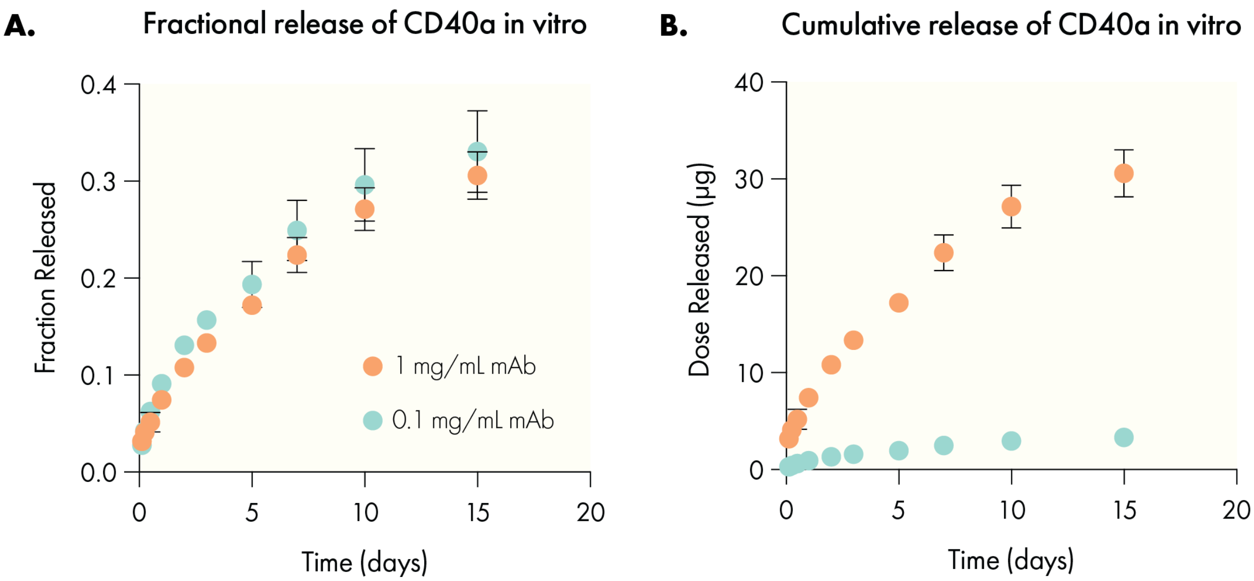

Supplemental Figure 3. Release kinetics of CD40 agonist antibody from PNP hydrogels under physiologically relevant *in vitro* conditions. (A) Sustained release profiles and similar timescales of release are observed from hydrogels despite order-of-magnitude difference in encapsulated cargo content. (B) Cumulative release indicates similar timeframes to release significantly different doses of CD40 agonist.

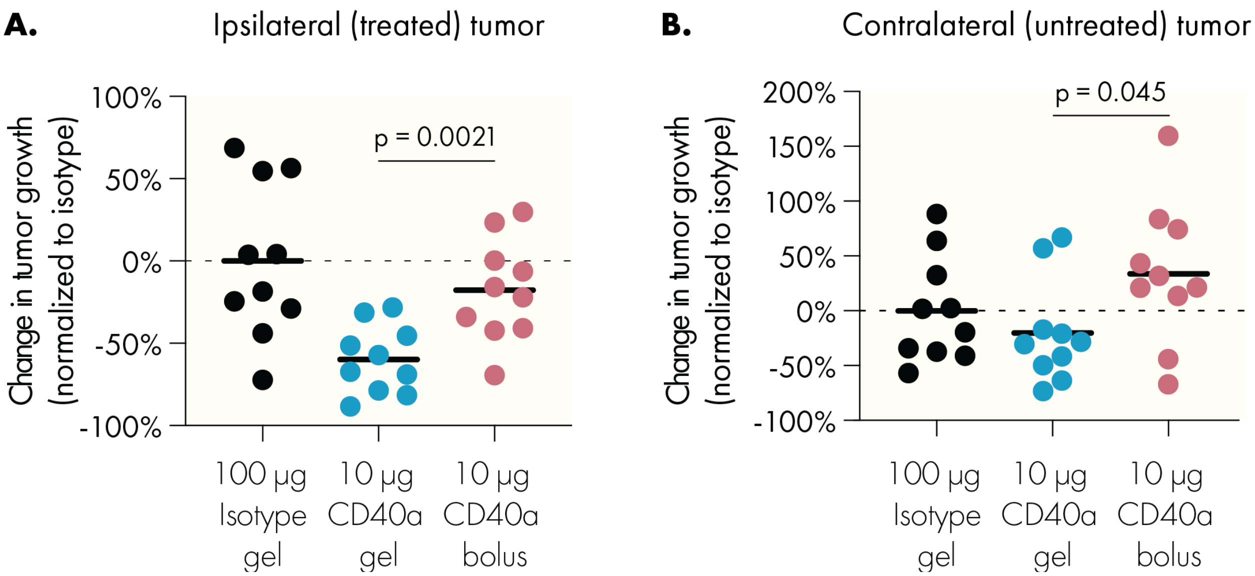

Supplemental Figure 4. Tumor growth inhibition and abscopal effects are influenced by CD40a administration route. Dual-flank tumor bearing mice were treated with a single dose of CD40a or a negative isotype control. (A) The ipsilateral, or treated, tumor exhibits growth inhibition relative to negative isotype controls, with hydrogel administration resulting in more profound growth inhibition. (B) The distant contralateral, or untreated, tumor growth is more inhibited in mice treated with CD40a hydrogels than mice treated with a locoregional dose-matched bolus of CD40a.

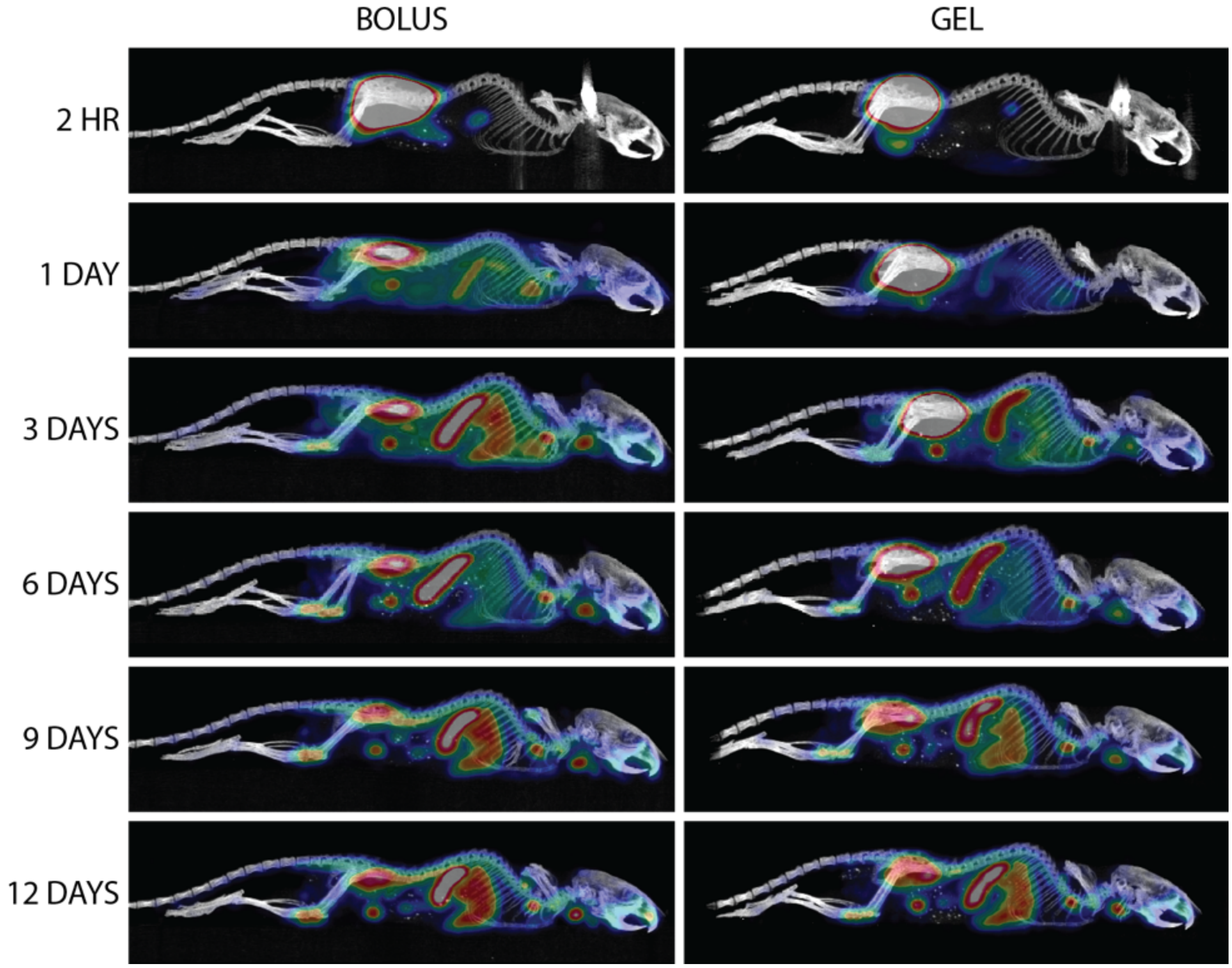

Supplemental Figure 5. Representative PET images comparing CD40a biodistribution over the course of 12 days after administration via local bolus or hydrogel.

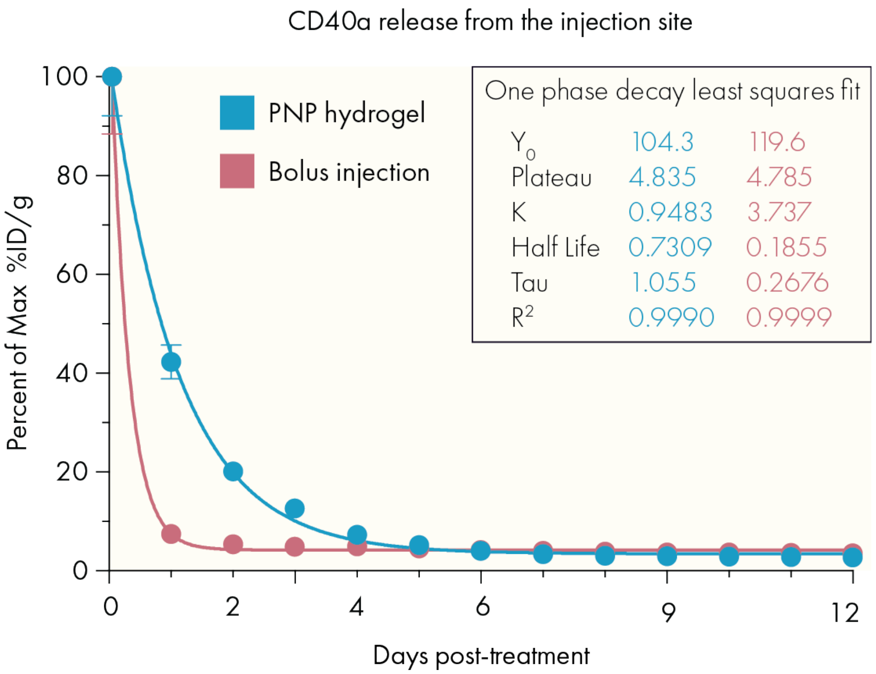

Supplemental Figure 6. Quantified CD40a concentration over time curves at the injection site from PET imaging.

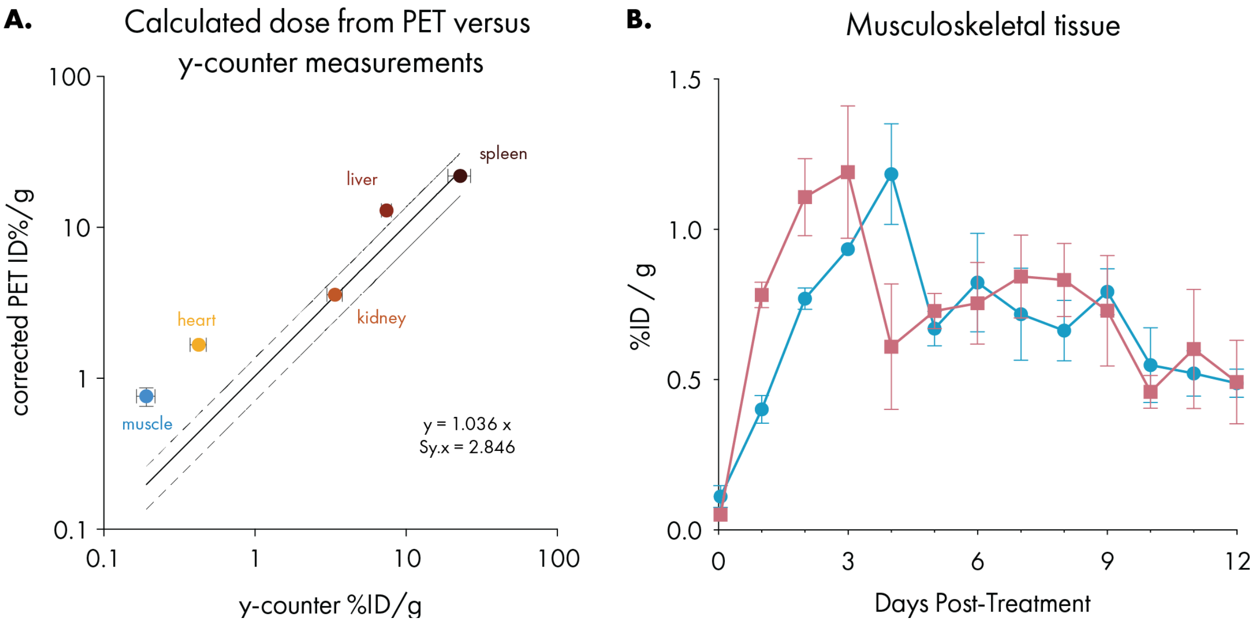

Supplemental Figure 7. Quality control of PET measurements and assessment of background from unbound radioisotope. (A) Biodistribution data calculated from PET scans compared to values measured from explanted organs using a gamma counter. (B) Unbound Zr^{89} bioaccumulates in bone tissues, and ROI were drawn over the thigh muscle and femur to assess background signal that could be attributed to unbound radioisotope. Signal in this tissue is below 1.5 %ID/g over the course of the study.

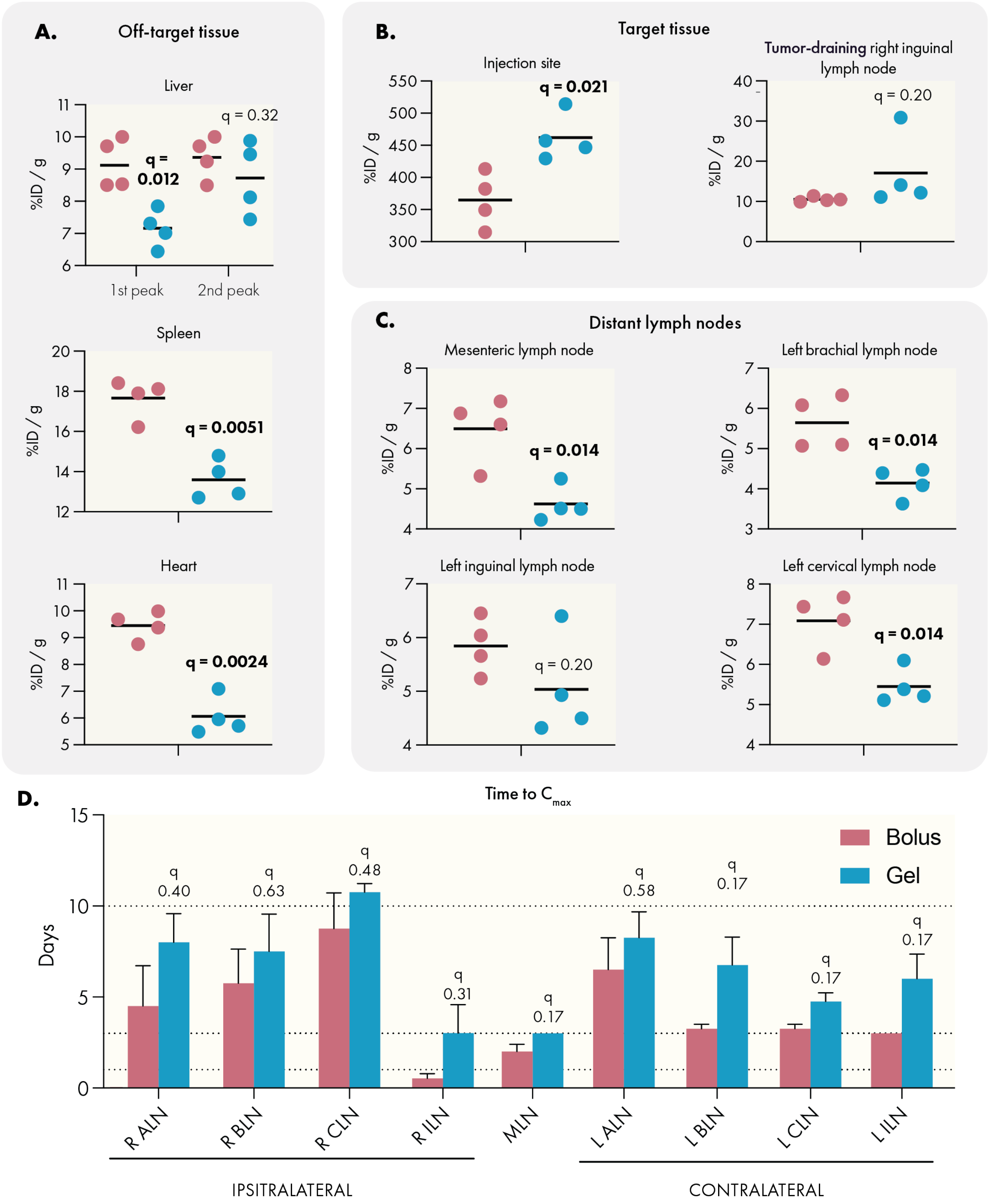

Supplemental Figure 8. Additional pharmacokinetic metrics for CD40a delivery, calculated from PET biodistribution study. (A) C_{max} values for off-target organs. (B) C_{max} values for target tissues. (C) C_{max} values for distant lymph nodes. (D) Mean time to C_{max} for lymph nodes, error bars represent SEM. N = 4 for each treatment.

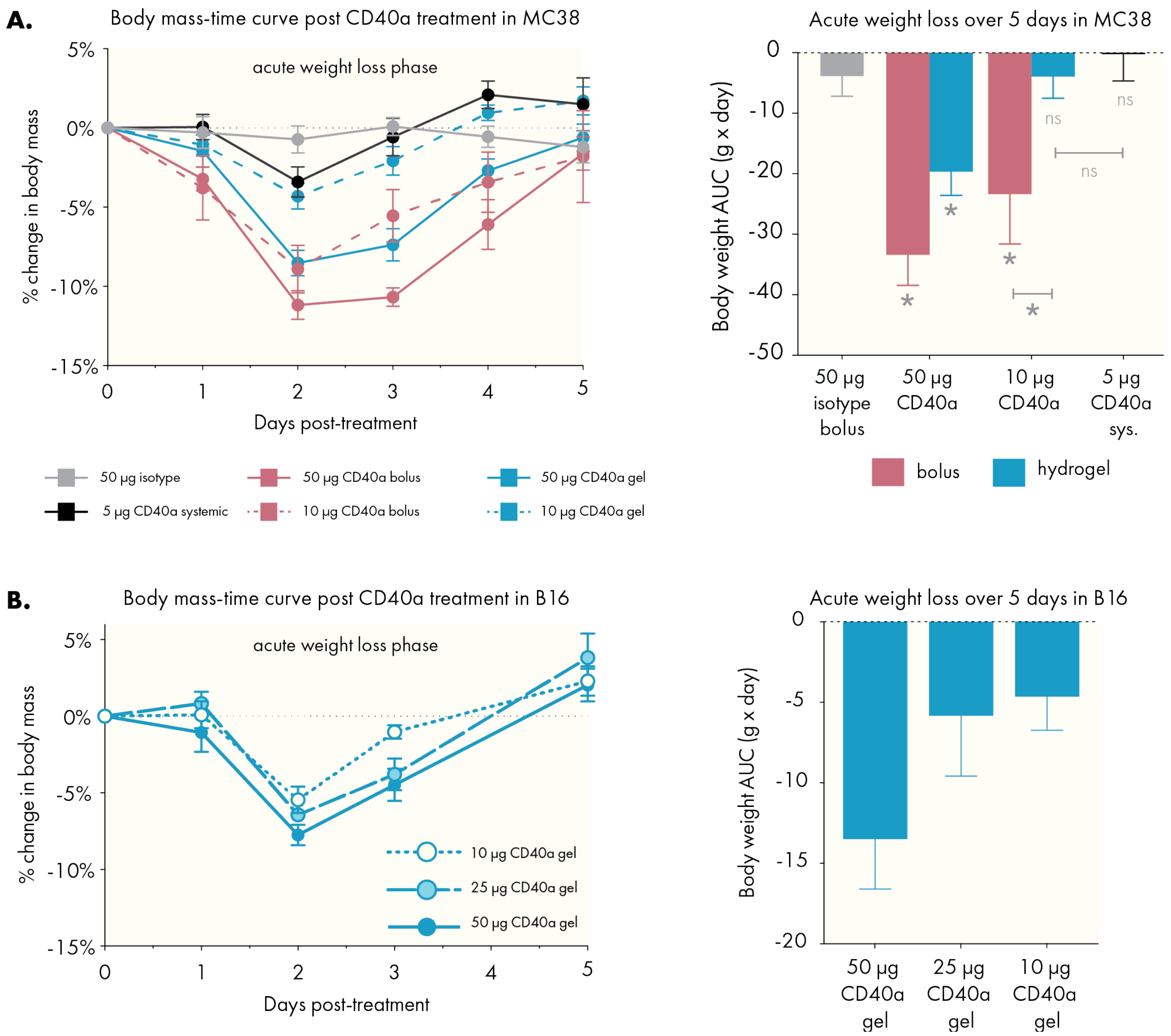

Supplemental Figure 9. Body weight-time curves for MC38 and B16 studies in mice.

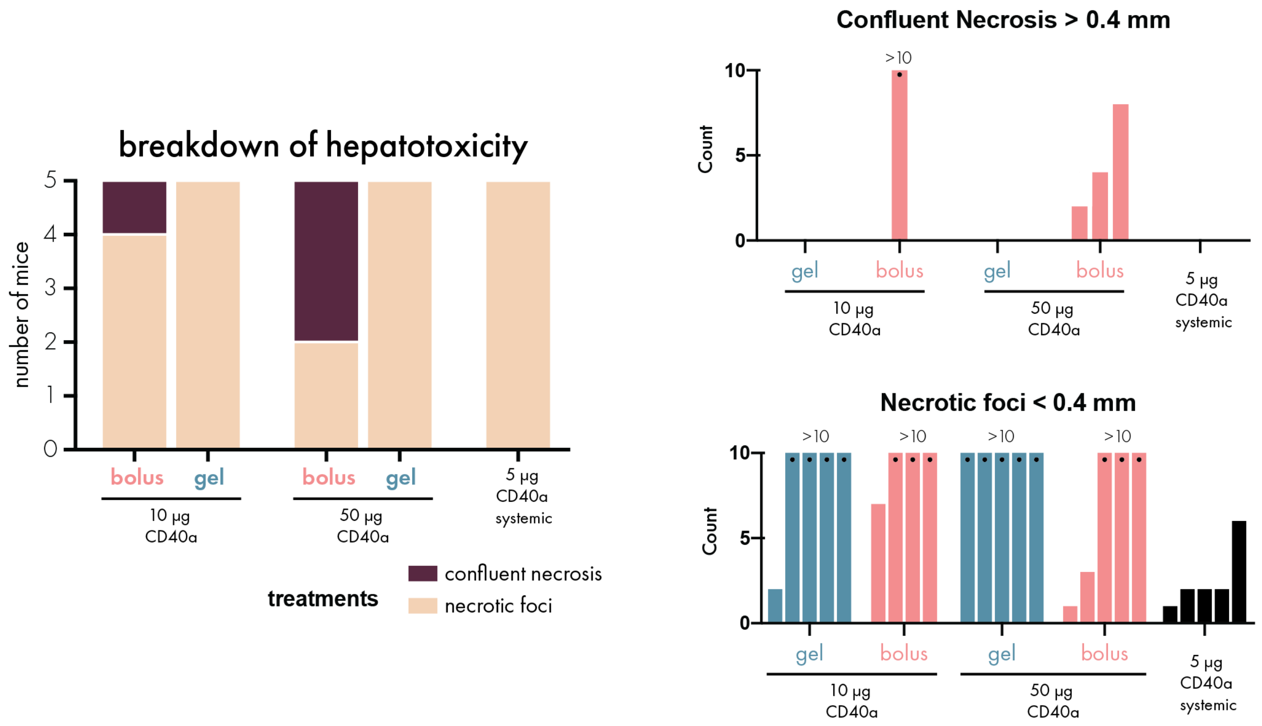

Supplemental Figure 10. Quantitative breakdown of liver toxicity determined from liver histology 72 hours after treatment with CD40a.

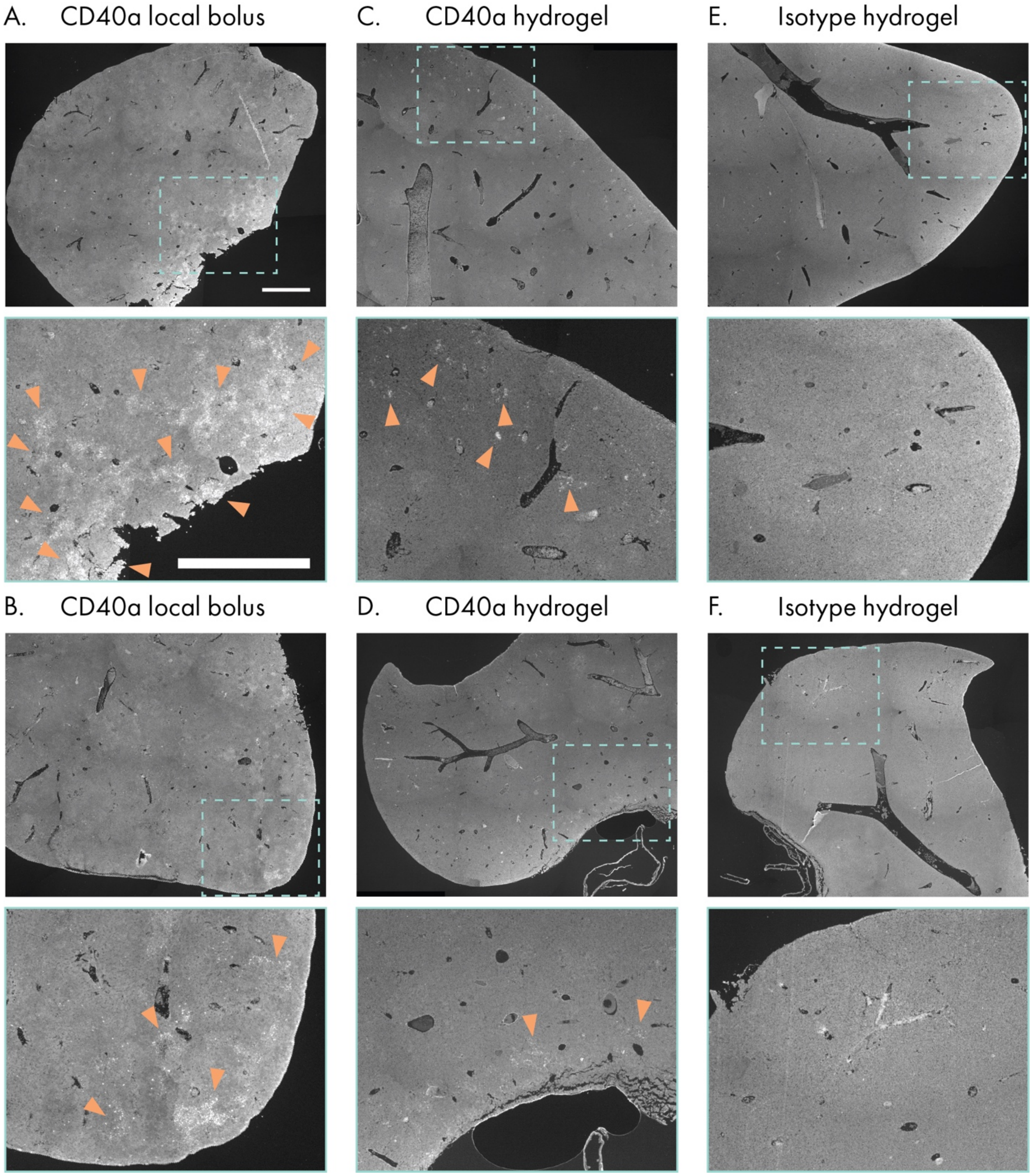

Supplemental Figure 11. Cleaved caspase 3 expression in liver tissues 72 hours after treatment. (A, B) Treated with 10 µg of CD40a as a local bolus. (C, D) Treated with 10 µg of CD40a in a hydrogel. (E, F) Treated with isotype control antibody loaded in a hydrogel. Scale bars in (A) indicate 1 mm in both the low magnification (top panels) or in the high magnification (low panels) images. Images within a treatment category are of different lobes of the liver.

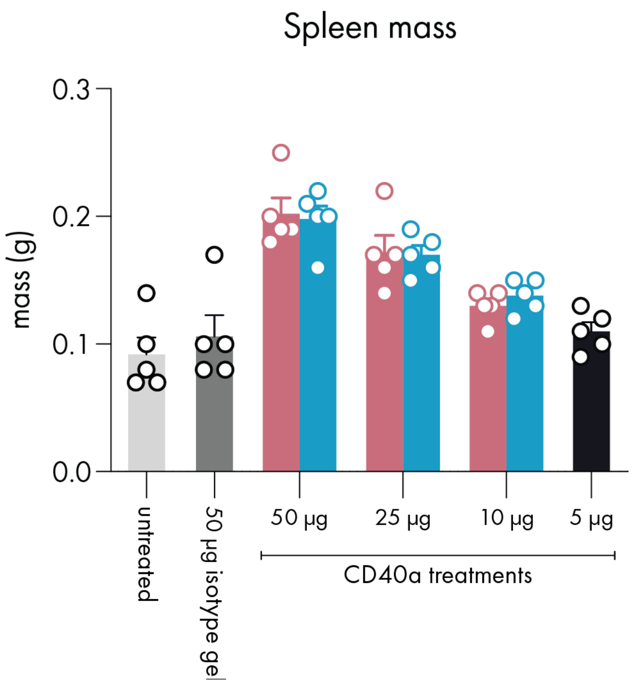

Supplemental Figure 12. Spleen mass at different doses in gel or bolus 72 hours after treatment.

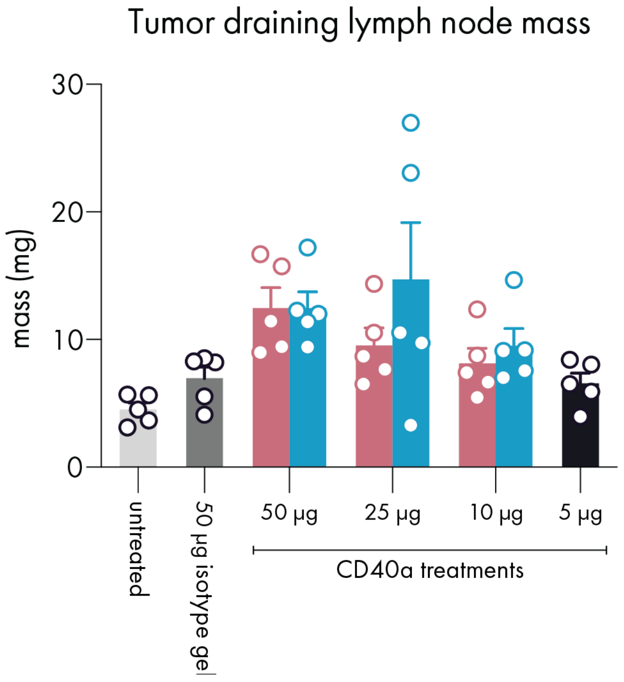

Supplemental Figure 13. Lymph node mass following different doses of CD40a in gel or bolus, 72 hours after treatment.

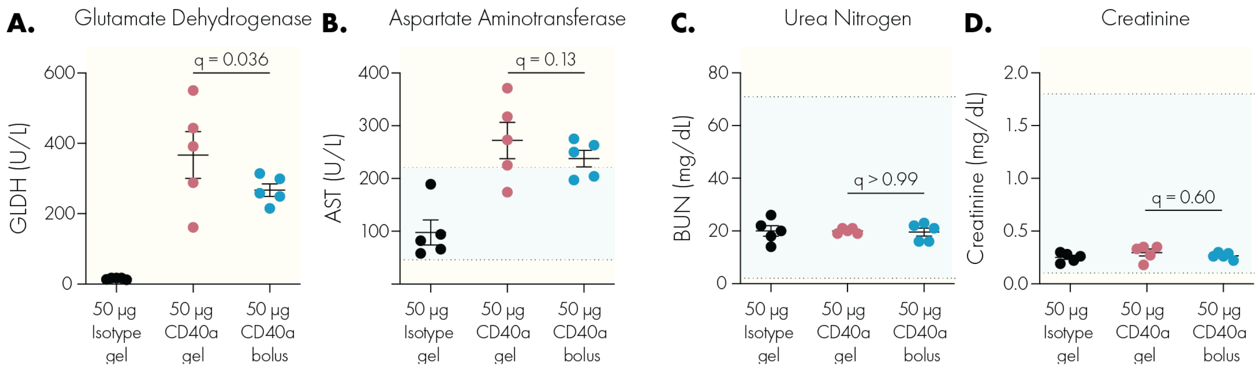

Supplemental Figure 14. Blood chemistry from tumor-bearing mice treated with CD40a via either local bolus or injectable hydrogel evaluating impact on hepatotoxicity and nephrotoxicity. (A) Glutamate dehydrogenase (GLDH), a marker of acute hepatoxicity. (B) Aspartate aminotransferase activity (AST), a marker for hepatoxicity. (C) Urea Nitrogen (BUN) assay and (D) creatinine levels as markers of nephrotoxicity. Reference ranges indicated in green where available from the literature.[1]

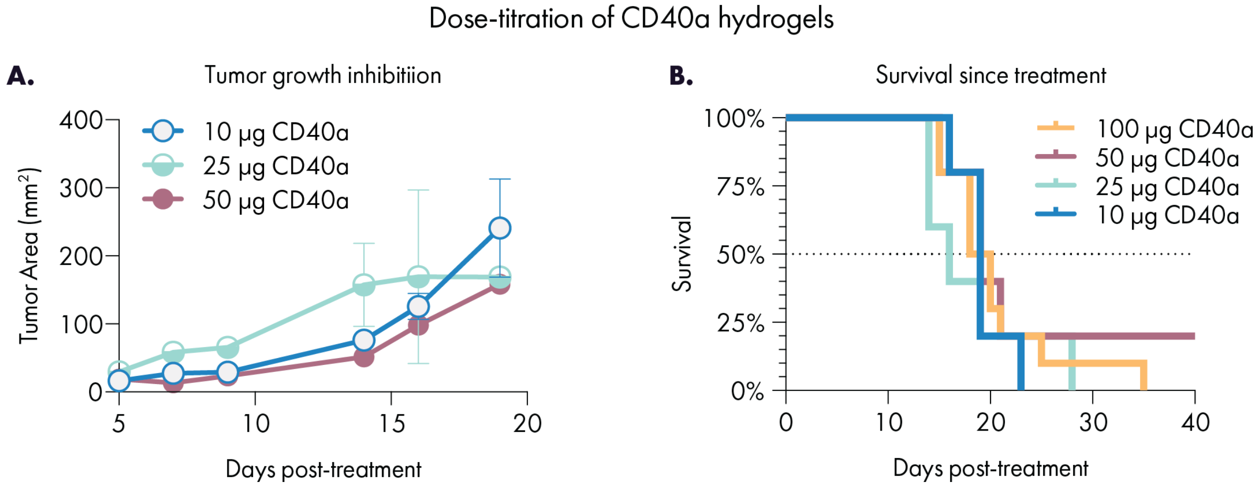

Supplemental Figure 15. Growth and Survival curves for dose titration in B16F10 using hydrogel delivery vehicles. N = 5 for 10, 25, and 50 µg groups and n = 10 for the 100 µg group.

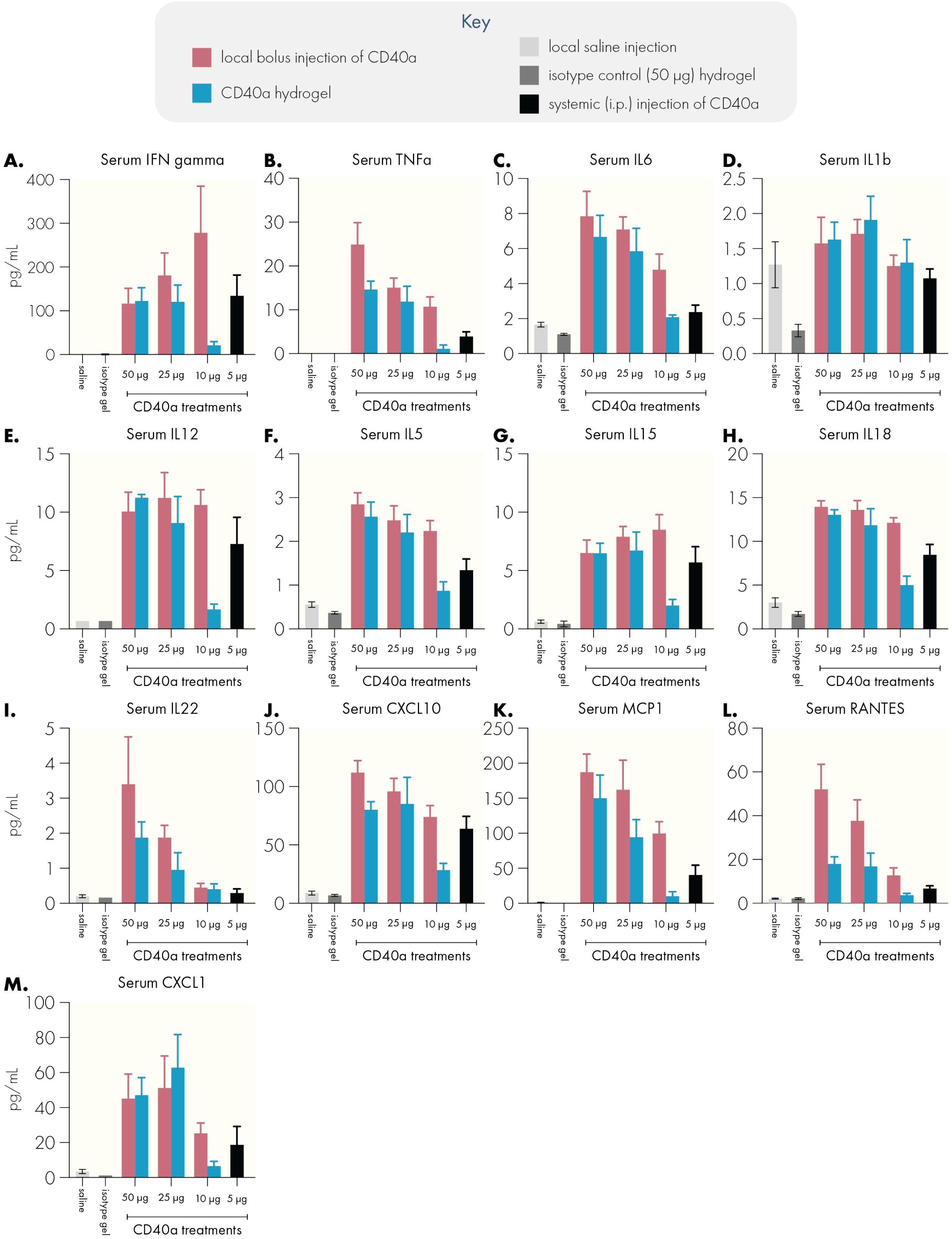

Supplemental Figure 16. Conversion of serum Luminex MFI signals to pg/mL serum concentrations based on standard curves for selected cytokines. For reasons discussed

in the main text, we provide these concentrations for comparison to other literature but center our analyses on more accurate assessments based on the corrected fluorescence measurements shown below.

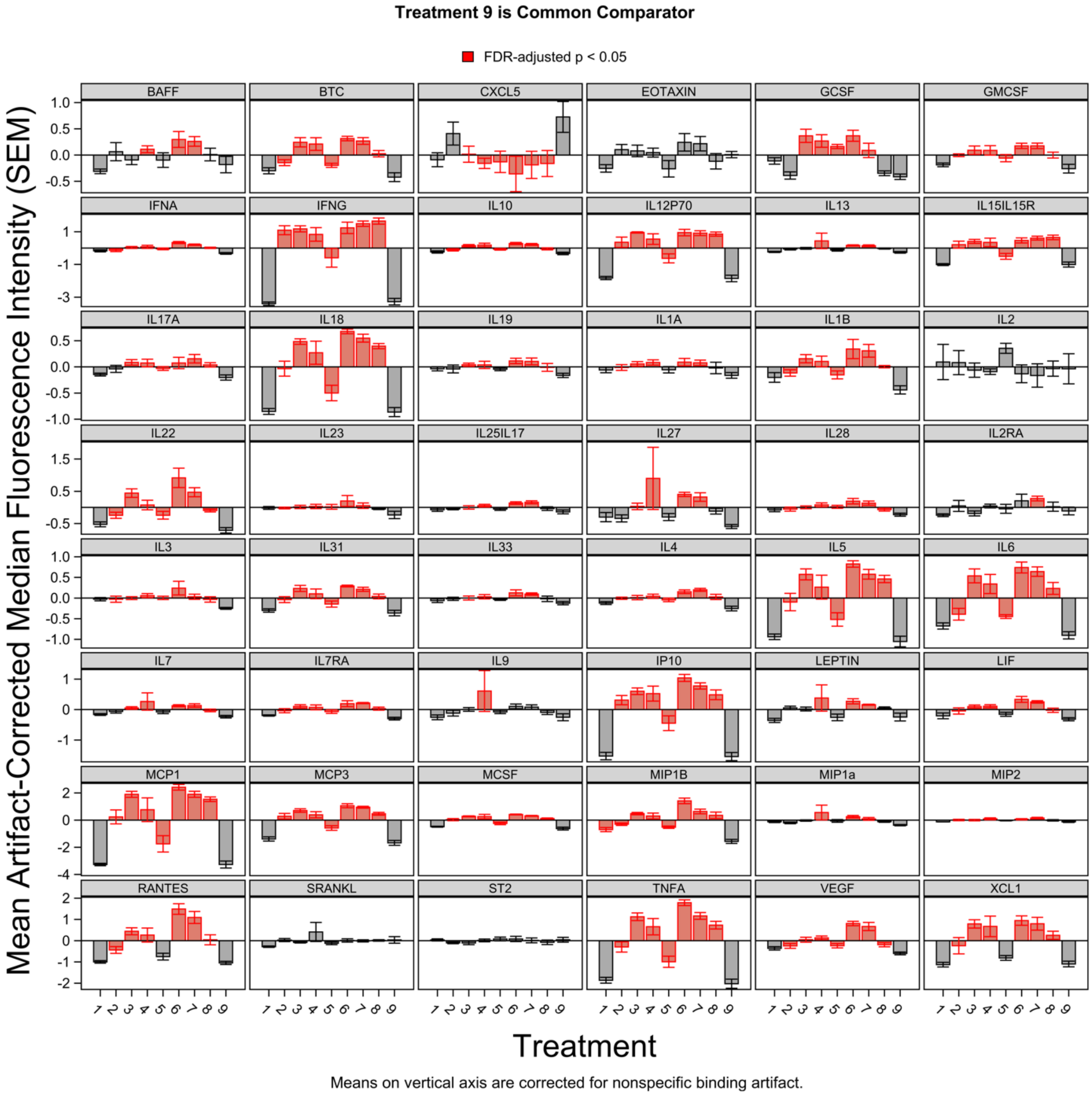

Supplemental Figure 17. Full artifact-corrected Luminex dataset for serum cytokine levels 24 hours after treatment. Treatment key: **1** = 50 µg isotype control; **2** = 5 µg CD40a systemic dose (maximum tolerated systemic dose); **3** = 50 µg CD40a in hydrogel; **4** = 25 µg CD40a in hydrogel; **5** = 10 µg CD40a in hydrogel; **6** = 50 µg CD40a as local bolus; **7** = 25 µg CD40a as local bolus; **8** = 10 µg CD40a as local bolus; **9** = untreated.

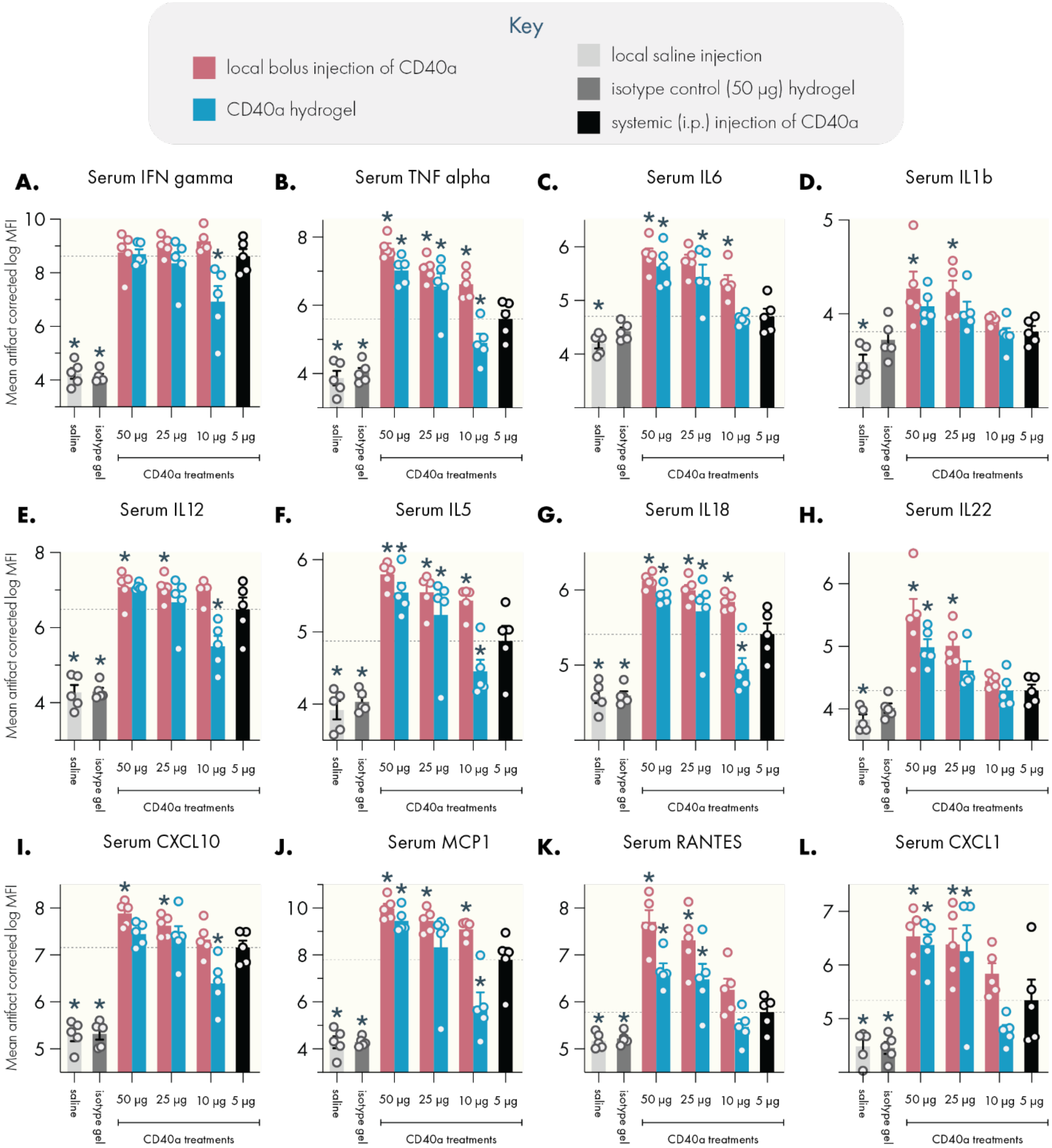

Supplemental Figure 18. Highlighted artifact-corrected serum cytokine data. Dotted line indicates mean value corresponding to the 5 µg systemic dose (maximum tolerated systemic dose). N = 5 for all groups, data represented as mean and SEM. * Denote a significant difference ($p < 0.05$) from the MTD systemic dose. Multiple testing error was controlled using the FDR approach (Q = 5%).

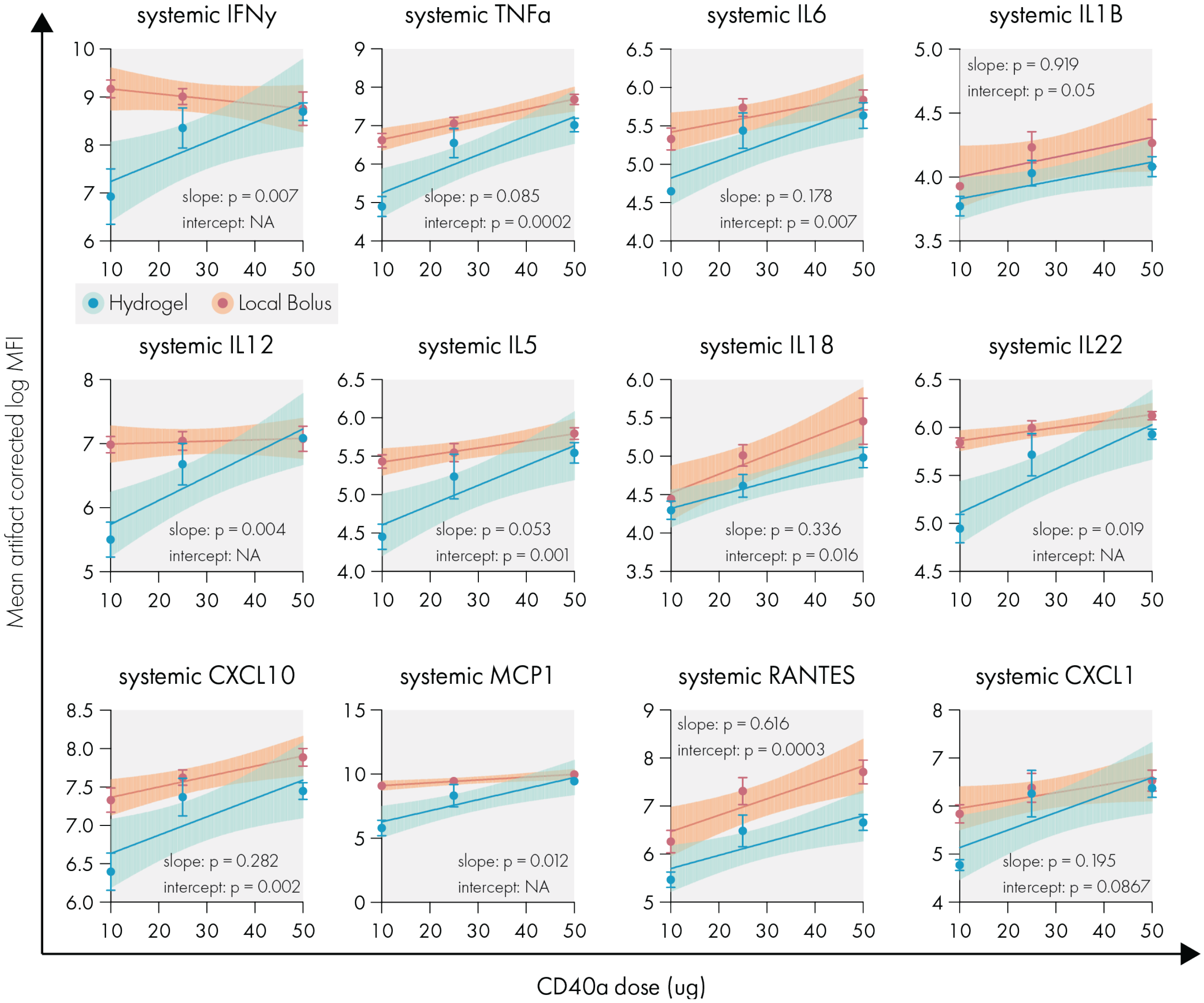

Supplemental Figure 19. Simple linear regression, with no constraints, of dose-serum cytokine response curves for individual cytokines. Shaded area represents 95% confidence interval, error bars indicate SEM. Statistical comparisons performed using built-in analysis in the linear regression functionality of GraphPad Prism.

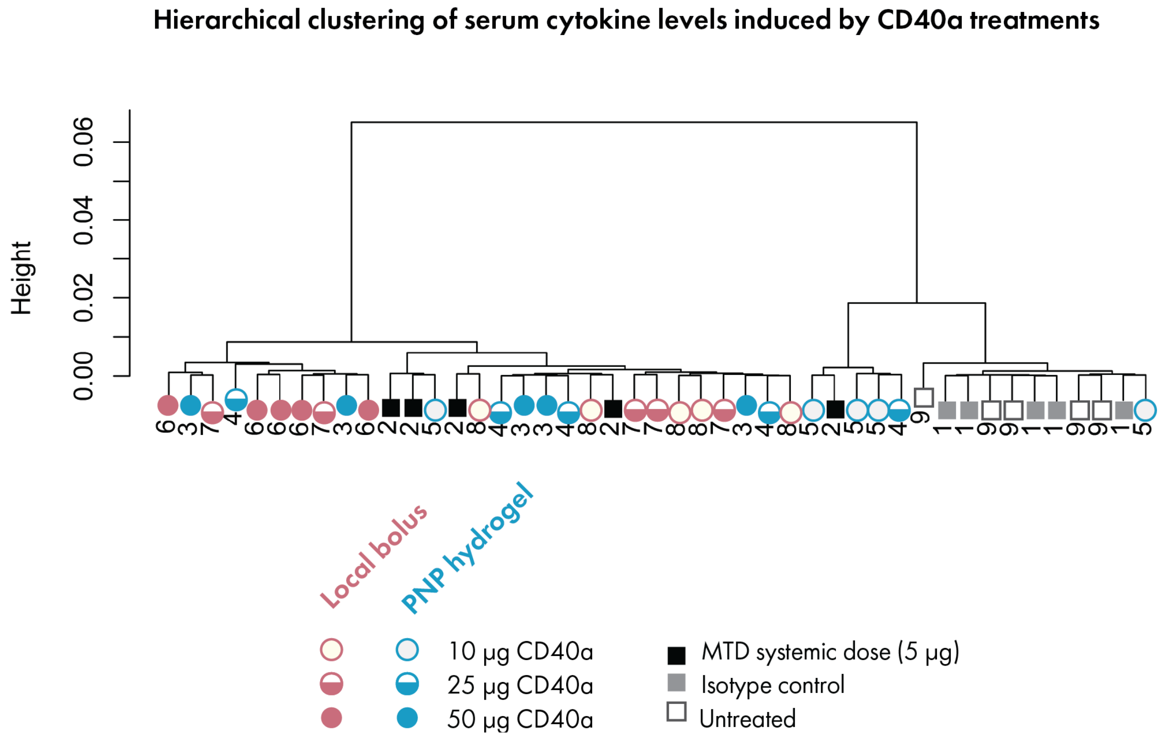

Supplemental Figure 20. Serum cytokine response dendrogram.

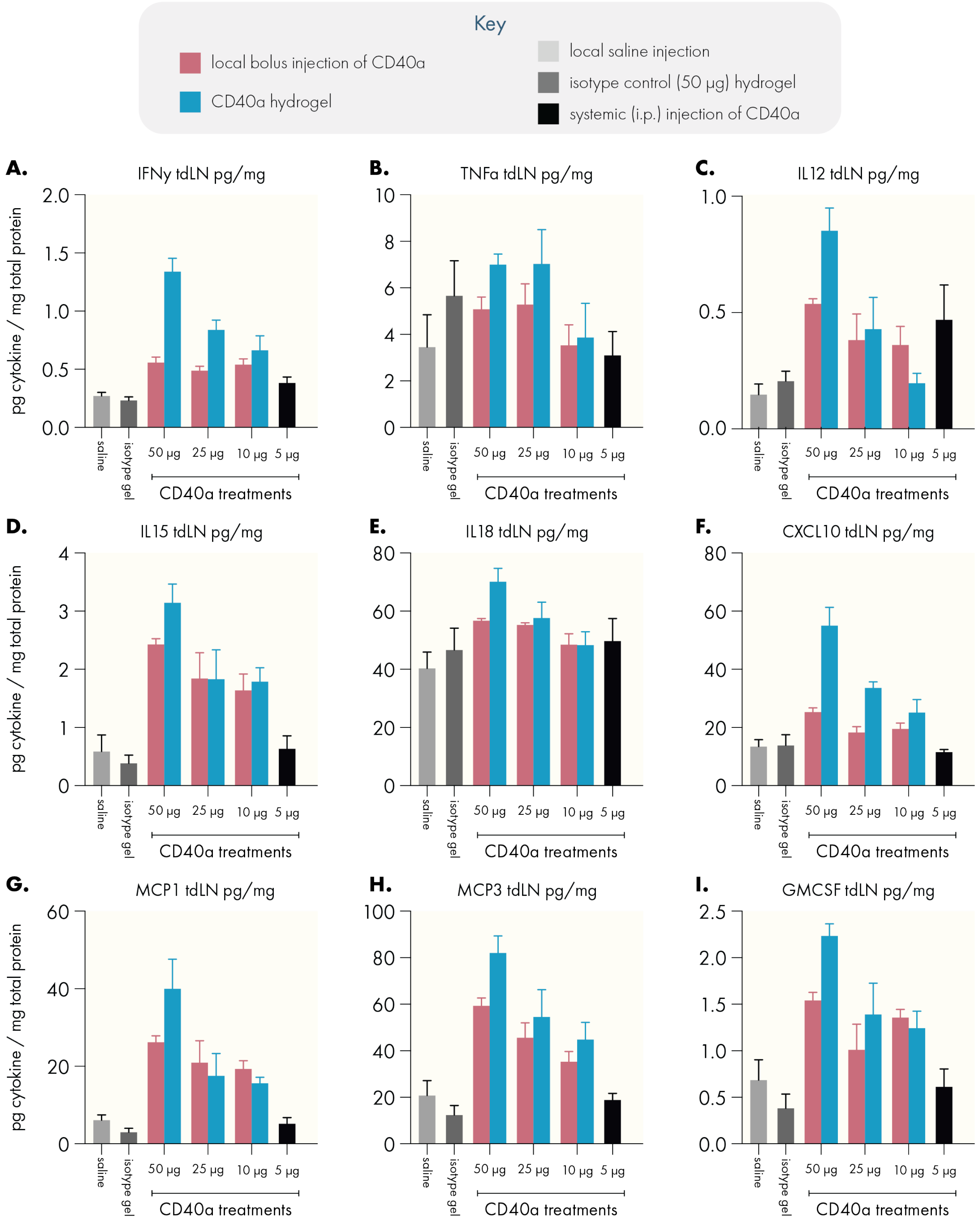

Supplemental Figure 21. Conversion of TdLN Luminex MFI signals to pg/mg total protein based on standard curves for selected cytokines. For reasons discussed in the main text, we provide these concentrations for comparison to other literature but center

our analyses on more accurate assessments based on the corrected fluorescence measurements shown below.

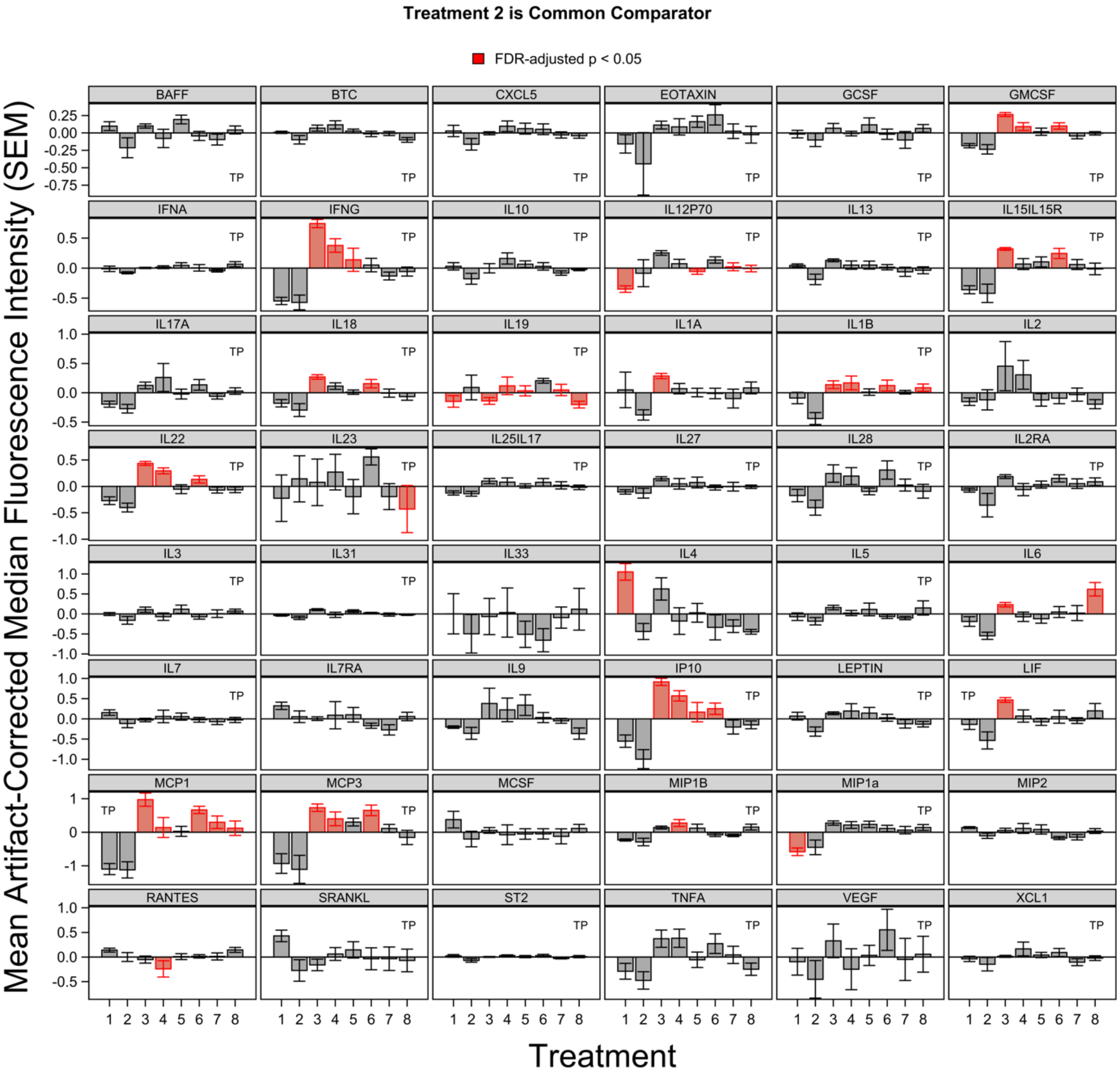

Means on vertical axis are corrected for plate and nonspecific binding artifacts.
TP = statistically significant correction for total protein.

Supplemental Figure 22. Full artifact-corrected Luminex dataset for tumor-draining lymph node cytokine levels 3 days after treatment. Treatment key: **1** = 50 µg isotype control; **2** = 5 µg CD40a systemic dose (maximum tolerated systemic dose); **3** = 50 µg CD40a in hydrogel; **4** = 25 µg CD40a in hydrogel; **5** = 10 µg CD40a in hydrogel; **6** = 50 µg CD40a as local bolus; **7** = 25 µg CD40a as local bolus; **8** = 10 µg CD40a as local bolus.

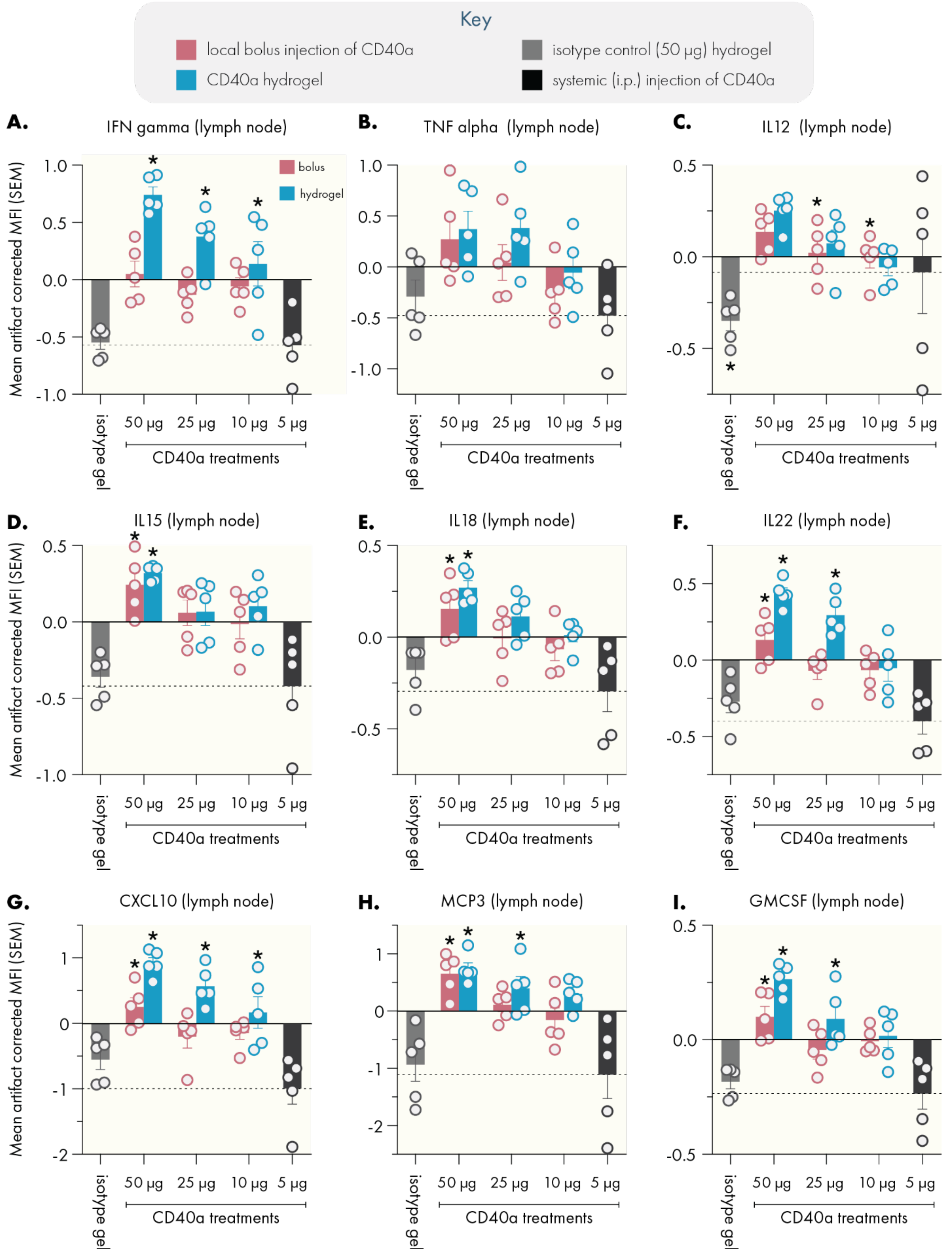

Supplemental Figure 23. Highlighted artifact-corrected lymph node cytokine data. Dotted line indicates mean value corresponding to the 5 μg systemic dose (maximum

tolerated systemic dose). N = 5 for all groups, data represented as mean and SEM. * Denote a significant difference ($p < 0.05$) from the MTD systemic dose. Multiple testing error was controlled using the FDR approach (Q = 5%).

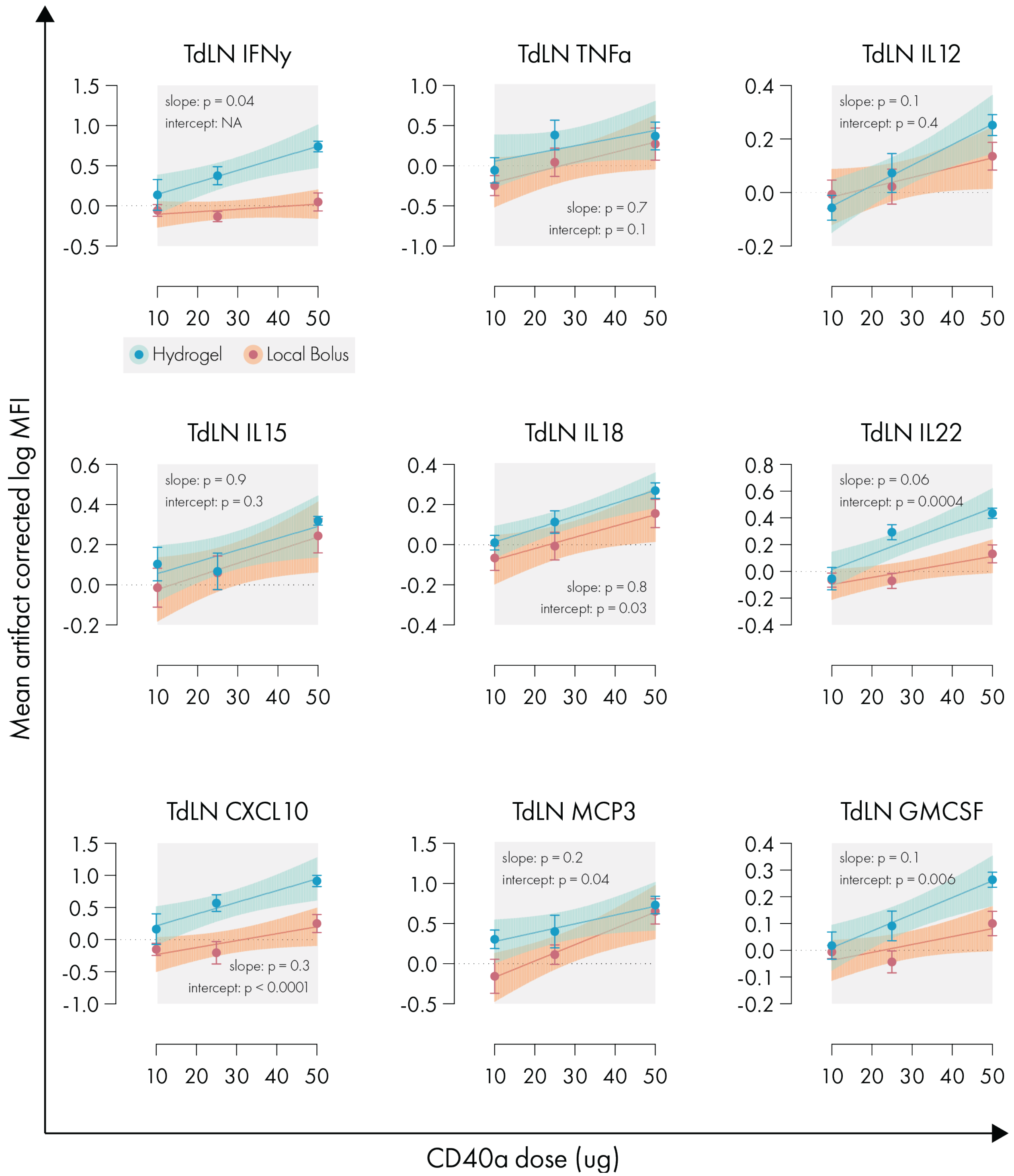

Supplemental Figure 24. Simple linear regression, with no constraints, of dose-TdLN cytokine response curves for individual cytokines. Shaded area represents 95% confidence interval, error bars indicate SEM. Statistical comparisons performed using built-in analysis in the linear regression functionality of GraphPad Prism.

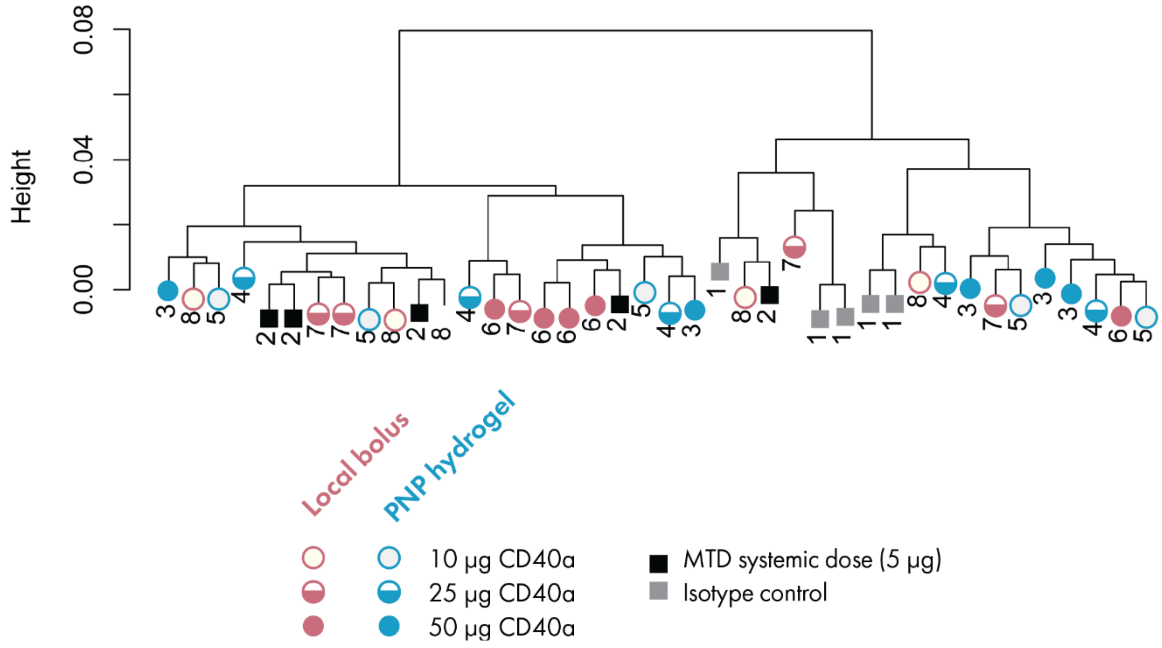

Supplemental Figure 25. TdLN cytokine response dendrogram.

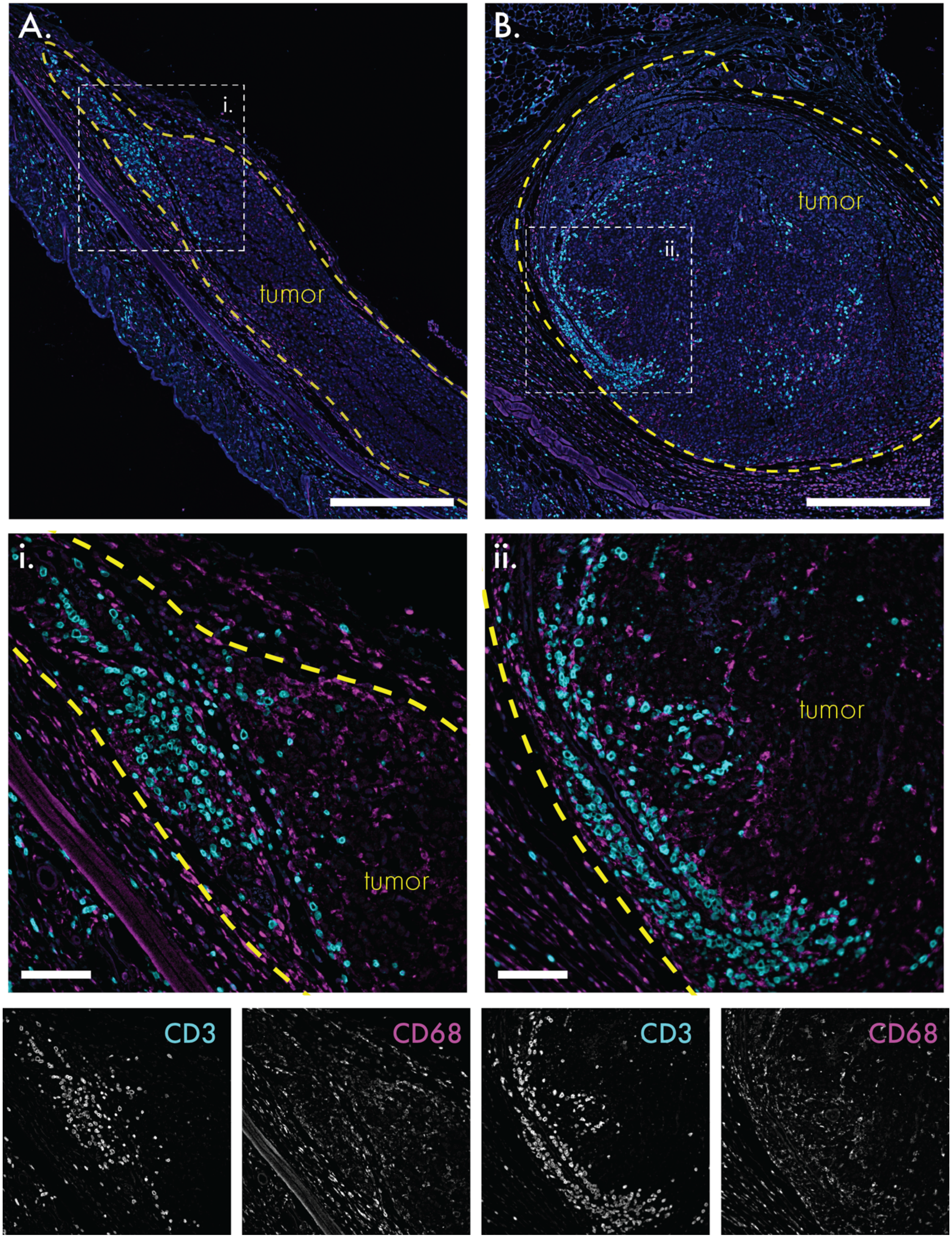

Supplemental Figure 26. Locoregional CD40a therapy transforms the tumor immune microenvironment into a more immunogenic state. B16 tumors were explanted for

immunohistochemistry 3 days after treatment and stained for the pan-T cell marker CD3 (cyan) and the macrophage marker CD68 (magenta). Blue indicates DAPI nuclear stain. (A) Tumor tissue treated with 10 μg dose of CD40a in a hydrogel. (B) Tumor tissue treated with 10 μg dose of CD40a as a local bolus. Tumor borders are indicated by the yellow dotted line. (i, ii, and iii) Magnification of CD3+ cell infiltrated zones as indicated by the white dotted lines in panels A-C. Single channel grayscale images of the CD3 and CD68 stains in the detailed view are provided below. Scale bars: A-B denote 500 microns; i-ii denote 100 microns.

References

[1] F. W. Quimby, R. H. Luong, *The Mouse in Biomedical Research* **2007**, 171, https://doi.org/10.1016/B978-012369454-6/50060-1.
